# Assessment of the incorporation of CNV surveillance into gene panel next-generation sequencing testing for inherited retinal diseases

**DOI:** 10.1136/jmedgenet-2017-104791

**Published:** 2017-10-26

**Authors:** Jamie M Ellingford, Bradley Horn, Christopher Campbell, Gavin Arno, Stephanie Barton, Catriona Tate, Sanjeev Bhaskar, Panagiotis I Sergouniotis, Rachel L Taylor, Keren J Carss, Lucy F L Raymond, Michel Michaelides, Simon C Ramsden, Andrew R Webster, Graeme C M Black

**Affiliations:** 1 Manchester Centre for Genomic Medicine, Manchester Academic Health Sciences Centre, Manchester University NHS Foundation Trust, St Mary’s Hospital, Manchester, UK; 2 Division of Evolution and Genomic Sciences, Neuroscience and Mental Health Domain, School of Biological Sciences, Faculty of Biology, Medicine and Health, University of Manchester, Manchester, UK; 3 Department of Genetics, UCL Institute of Ophthalmology, London, UK; 4 Congenica, Wellcome Genome Campus, Hinxton, Cambridge, UK; 5 Department of Haematology, University of Cambridge NHS Blood and Transplant Centre, Cambridge, UK; 6 Department of NIHR BioResource – Rare Diseases, Cambridge University Hospitals NHS Foundation Trust, Cambridge Biomedical Campus, Cambridge, UK; 7 Department of Medical Genetics, Cambridge Institute for Medical Research, University of Cambridge, Cambridge, UK; 8 Moorfields Eye Hospital NHS Foundation Trust, London, UK

**Keywords:** copy-number variation, next-generation sequencing, molecular genetics, inherited retinal disease

## Abstract

**Background:**

Diagnostic use of gene panel next-generation sequencing (NGS) techniques is commonplace for individuals with inherited retinal dystrophies (IRDs), a highly genetically heterogeneous group of disorders. However, these techniques have often failed to capture the complete spectrum of genomic variation causing IRD, including CNVs. This study assessed the applicability of introducing CNV surveillance into first-tier diagnostic gene panel NGS services for IRD.

**Methods:**

Three read-depth algorithms were applied to gene panel NGS data sets for 550 referred individuals, and informatics strategies used for quality assurance and CNV filtering. CNV events were confirmed and reported to referring clinicians through an accredited diagnostic laboratory.

**Results:**

We confirmed the presence of 33 deletions and 11 duplications, determining these findings to contribute to the confirmed or provisional molecular diagnosis of IRD for 25 individuals. We show that at least 7% of individuals referred for diagnostic testing for IRD have a CNV within genes relevant to their clinical diagnosis, and determined a positive predictive value of 79% for the employed CNV filtering techniques.

**Conclusion:**

Incorporation of CNV analysis increases diagnostic yield of gene panel NGS diagnostic tests for IRD, increases clarity in diagnostic reporting and expands the spectrum of known disease-causing mutations.

## Introduction

Inherited retinal dystrophies (IRDs) are a set of genetic disorders that have a diverse pathogenesis and are characterised by extreme genetic and clinical heterogeneity.^1 2^ They are the leading cause of blindness in working-age adults in the UK,^3^ and are present in a range of multisystemic disorders, such as Usher syndrome and Senior-Loken syndrome. Identifying the genetic basis of IRDs can greatly assist the clinical diagnosis, counselling, treatment and management received by referred individuals.^4^ As a result, a number of genomic diagnostic tests are available for individuals with IRD, including SNP microarrays, direct sequencing approaches, array comparative genomic hybridisation (array CGH) and high-throughput sequencing (commonly referred to as next-generation sequencing, *NGS*).^5^ Despite the emergence of whole exome^6^ and whole genome NGS approaches,^7^ gene panel NGS approaches remain a major first-tier diagnostic test. This is due to their affordability, specificity, high coverage and proven capability to characterise disease-causing single nucleotide variations (SNVs) and small insertion and deletion events (indels).^8 9^ However, the informatics techniques used to detect genetic variation from gene panel NGS diagnostic services have often failed to truly characterise the spectrum of disease-causing variation within the IRDs, including the relative contribution of large structural variation and CNV.

CNVs result in the gain or loss of genomic material and are known to cause IRD.^10^ However, the insertion and breakpoints of CNVs are often deeply intronic or intergenic, and as a result are not captured by gene panel NGS approaches employed in diagnostic environments, which focus primarily on protein-coding regions and proven pathogenic intronic variants. This creates limitations in the types of variant detection algorithms that can be applied to gene panel NGS data sets to detect CNVs.^11^ Read-depth approaches for the surveillance of CNVs, with complementary quality assurance parameters, have recently been applied to gene panel NGS data sets in a diagnostic context.^12–14^ Moreover, recent studies investigating the role of CNVs in IRDs have identified an enrichment of disease-causing CNVs among individuals without a genetic diagnosis through gene panel NGS techniques,^7 15^ and demonstrated the capability of high-resolution array CGH,^16^ whole exome sequencing (WES)^17^ and whole genome sequencing (WGS)^7 18^ to identify CNVs within and encompassing these surveyed genes. While the potential to identify CNVs from gene panel NGS data sets for IRD has been shown,^19^ this analysis is yet to be extended to a large cohort of individuals using comprehensive NGS gene panels generated through accredited diagnostic services. As such, knowledge of the relative benefits and limitations of introducing CNV surveillance into first-tier diagnostic gene panel NGS services for IRD remains limited.

In this study, we have expanded the assessment of gene panel NGS diagnostic data sets to include CNV analysis among a large cohort of 550 individuals with IRD. Through comparison to WGS samples, we demonstrate the advantages and limitations of this approach, and illustrate an informatics workflow for the analysis of CNVs identified from gene panel NGS data sets. Taken together, incorporation of CNV analysis increases the diagnostic yield of a major first-tier diagnostic test for IRD, increases clarity in diagnostic reporting and expands the spectrum of known disease-causing mutations.

## Materials and methods

### Recruitment of patients for CNV analysis

We performed CNV analyses for 550 individuals with clinical indications of IRD. All individuals provided consent for the comprehensive analysis of variation in genes known as a cause of IRD and were referred for diagnostic genetic testing by clinicians at Manchester Royal Eye Hospital and Moorfields Eye Hospital, London.

### Generation of gene panel NGS data sets

DNA was extracted from the peripheral blood of referred individuals and enriched for specified regions of the genome using an Agilent SureSelect Custom Design target-enrichment kit (Agilent, Santa Clara, California, USA). Enrichment kits were designed to capture known pathogenic intronic variants and the protein-coding regions ±50 nucleotides of selected National Center for Biotechnology Information (NCBI) RefSeq transcripts for 105 or 180 genes known as a cause of IRD (online supplementary table S1). Full details of the genes and analysis techniques used during the 105-gene diagnostic testing procedure (referred to as v2) can be found in Ellingford *et al* 9 and through the UK Genetic Testing Network (https://ukgtn.nhs.uk/find-a-test/search-by-disorder-gene/retinal-degeneration-105-gene-panel-568/). The 180-gene panel (referred to as v3) represents an expanded iteration of this diagnostic service within the UK National Health Service, with the additional inclusion of enrichment baits to capture (1) selected pathogenic intronic variants; and (2) additional genes known as a cause of IRD, including newly identified genes and genes known as a cause of congenital stationary night blindness. After enrichment, samples were pooled using unique barcode identifiers, and paired-end high-throughput sequencing was performed using the Illumina HiSeq 2000/2500.

10.1136/jmedgenet-2017-104791.supp1 Supplementary file 1

### Detection of CNVs from gene panel NGS data sets using ExomeDepth

Sequencing reads were demultiplexed with CASAVA V.1.8.2 and aligned to the *hg19* reference genome using Burrows-Wheeler Aligner short read (V.0.6.2) software.^20^ Duplicate reads were removed using SAMtools V.0.1.18 before variant calling was performed. We have described the methodology employed for the detection and clinical analysis of SNVs and indels previously.^9^ CNV detection was performed using standard parameters for ExomeDepth V.1.1.6.^21^ ExomeDepth was presented with sets of aligned and non-duplicate sequencing reads in a binary sequence alignment/map (BAM) file format that were matched by gender and by the enrichment kit used, and had been generated for unrelated individuals with IRD referred for diagnostic testing (online supplementary table S2).

### Informatics filtering strategies

**Figure 1:**
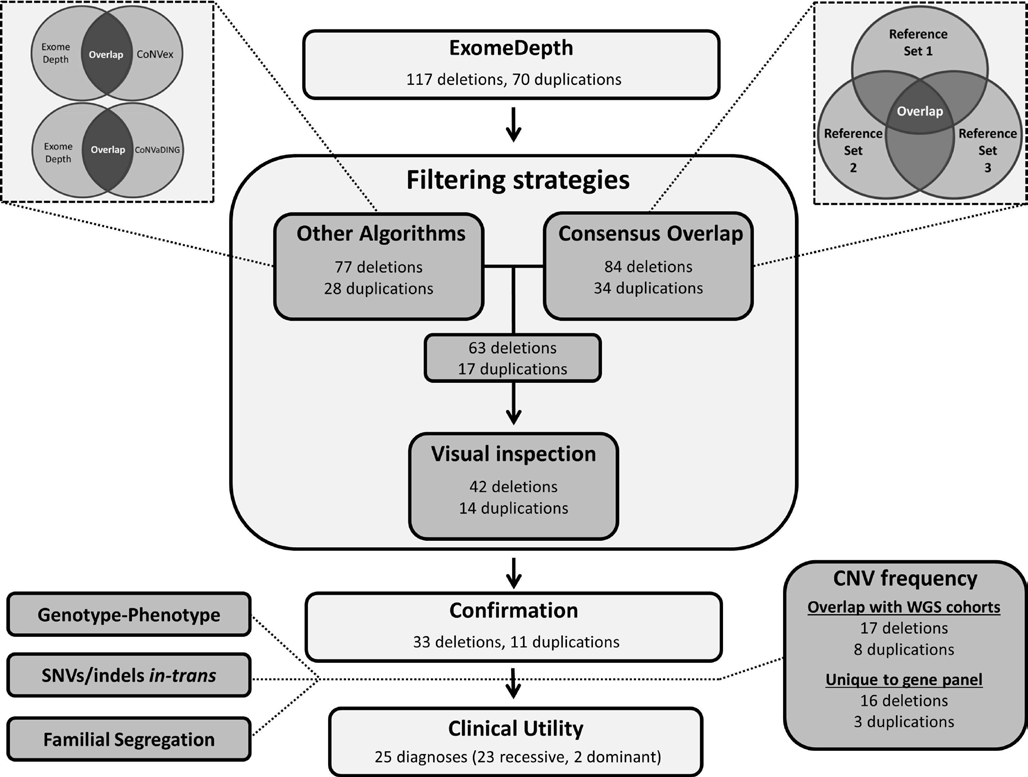
Informatics strategies used to filter CNVs identified by ExomeDepth. Taken together, these strategies had a positive predictive value of 79%. SNV, single nucleotide variation; WGS, whole genome sequencing.

We used three distinct strategies to limit the number of potential false-positive CNV events identified by ExomeDepth (figure 1). Events that were analysed in a clinical context were all (1) identified against three independent reference sets using ExomeDepth, (2) identified by at least one other CNV software tool (CoNVex,^22^ CoNVaDING^12^ or both) and (3) visually inspected using the ExomeDepth graphical package.

We first limited our analysis of CNV events to those that had been identified by ExomeDepth in comparison to three mutually exclusive reference sets of samples. For each tested individual we created three randomly selected and non-overlapping groups of 30 individuals matched by their gender and the enrichment kit used and presented these to the ExomeDepth algorithm. The overlap between the three reference sets was calculated using bedtools V.2.25.0 intersect. Second, we performed CNV calling using two other publicly available CNV detection algorithms (CoNVex and CoNVaDING). Both algorithms were presented with aligned and non-duplicate sequencing reads in a BAM file format for large groups of individuals matched by gender and the enrichment kit used (as described in online supplementary table S2), and CNV calling was performed using standard parameters for each of these tools. We compared CNV events identified by CoNVex and CoNVaDING with those that had been identified by ExomeDepth using bedtools V.2.25.0 intersect, and included all events identified by ExomeDepth and at least one other CNV detection tool. We limited our third stage of analysis, visual inspection, to those events that were identified against three reference sets using ExomeDepth and by at least one additional CNV detection tool. Visual inspection included an assessment of the consistency of calculated read ratios across all exons within implicated genes, the extent of variation within the selected reference samples for each exon, the nature of the exon CNV status across the cohort and the continuity of abnormal CNV exons within the implicated gene.

### Clinical analysis of CNV events

CNVs were interpreted alongside SNVs and indels that had been detected through routine gene panel NGS diagnostic techniques, as described previously.^9^ For each individual, variants were categorised in accordance with the American College of Medical Genetics and Genomics (ACMG) guidelines,^23^ and pathogenic/likely pathogenic variants in a disease-causing state were determined to confirm or provisionally confirm a molecular diagnosis of IRD. CNV frequency estimations were calculated through comparison to 682 WGS data sets for individuals with clinical indications of IRD. Six hundred and five samples were generated using Illumina sequencing chemistry as part of the National Institute for Health Research (NIHR) BioResource Rare Diseases project,^18^ and the Manta and Canvas software algorithms were used to detect CNVs.^24 25^ Seventy-seven samples were generated using Complete Genomics sequencing chemistry,^26^ with CNVs identified using the Complete Genomics V.2.5 variant calling pipeline.^27^ Both of these strategies incorporate an assessment of sequencing read depth, an assessment of the read insert sizes and an assessment of sequencing read composition to identify CNV breakpoints/insertion points.

### Confirmation of identified CNVs

CNVs were confirmed as present before they were reported to referring clinicians. Where kits designed and created by MRC-Holland (Amsterdam, The Netherlands) were available, we carried out multiplex ligation-dependent probe amplification (MLPA) assays. In the absence of a suitable MLPA kit, we validated CNVs using a digital droplet PCR or a quantitative fluorescence methodology, as described previously.^14^

### Estimating accuracy for CNV identification

To ensure that the NGS data surveyed were appropriate for CNV surveillance, we calculated a series of sequencing coverage metrics. We have provided a full description of these calculated metrics and their utility previously,^14^ and these included (1) NGS coverage and normalised coverage for surveyed exons, (2) levels of insufficient coverage (<50 unique NGS reads) for surveyed nucleotides and exons, and (3) intersample variability, defined as the coefficient of variation of normalised NGS coverage across samples selected as the reference set by ExomeDepth.

## Results

### CNV identification and filtering strategies

We performed CNV calling for 550 individuals with IRD using gene panel NGS data sets generated through diagnostic testing in a clinically accredited laboratory (197 v2 gene panel, 105 genes; 353 v3 gene panel, 180 genes). CNV surveillance was performed using ExomeDepth V.1.1.6. for four groups of individuals matched by their gender and the enrichment kit used during gene panel NGS (online supplementary table S2). In total, we identified 117 potential deletion events and 70 potential duplications through ExomeDepth  (online supplementary table S3). This equated to an average of one CNV event per three individuals tested (min=0, max=16), although we observed a trend of no CNVs identified for most samples (n=429) and more than one CNV identified in few samples (n=23; online supplementary figure S1). We applied three distinct strategies for CNV filtering (see online supplementary methods and results) in order to identify true CNV events, and these analyses identified 56 CNV events (30% of the original 187) for further confirmation and clinical analysis (figure 1). To assess the accuracy of informatics filtering approaches, 13 events that were excluded through comparison to other CNV detection algorithms were also selected for further confirmation (online supplementary results).

### Estimating accuracy for CNV identification

Through previous investigations we have identified that the level of NGS coverage in tested samples and the extent of variation in NGS coverage across selected reference samples (intersample variability) are both key influencers of the accuracy of ExomeDepth applied to gene panel NGS data sets. In total, we surveyed 1 267 742 exons for CNVs (1590 exons in 197 cases and 2704 exons in 353 cases), with an average of 2389 unique NGS reads generated per exon (min=0, max=202 357, median=1579, SD=4013.7). We observed that >50 unique NGS reads were generated for all the nucleotides included within 99.2% (n=1 257 794) of the surveyed exons, although we were unable to accurately survey the CNV status for eight exons included within the v2 panel (105 genes) due to consistently poor coverage across the cohort (online supplementary table S4). Consistently poor coverage was not observed across individuals surveyed through the newer v3 gene panel (180 genes; online supplementary table S4).

The average normalised NGS coverage profiles for each exon were calculated, and extensive variability was observed across the complete cohort, with average intersample variability values per exon of 21.1% (n, exons=313 230) and 22.2% (n, exons=954 512) for the v2 and v3 gene panels, respectively (online supplementary figure S2). Intersample variation was reduced to 5.83% (n exons=1 224 686, median=5.25%, SD=3.28%), when observations were limited to the extent of variation among samples selected as the reference set by ExomeDepth for each tested sample. There were 43 056 exons excluded from this analysis due to the selection of a solitary sample as the reference set by ExomeDepth (n=41 512) or as a result of consistently poor coverage (n=1544). In comparison to previously published simulation data sets,^14^ 95% and 99% of the surveyed exons are consistent with an accuracy for single exon deletions of 98.7% and 98.2%, respectively (online supplementary figure S3).

### Confirmation of CNVs and clinical outcomes

We confirmed 44/56 CNV events through orthogonal techniques, determining a positive predictive value (PPV) of 79% for the informatics filtering strategies employed in this study (figure 1, online supplementary results). Expanding confirmations to also include 13 events excluded through comparison to other CNV detection algorithms confirmed the presence of a single likely benign duplication event in *NPHP1* (*14016366*; NM_000272.3:c.(?_−1)_(*1_?)dup) but reduced the PPV to 65.2% (45/69). In confirming these findings, we determined a molecular diagnosis or a provisional molecular diagnosis for 25 individuals and additional findings that did not account for a molecular diagnosis for 18 individuals (table 1). These results were obtained after full appraisal of the clinical indication of IRD for the referred individual and the analysis of SNVs and small indels from routine gene panel NGS testing. Of note, a single individual was confirmed with two independent heterozygous CNV events, neither of which was determined to account for a molecular diagnosis (*13009597*; table 1). Routine testing identified a pathogenic missense variant in *SNRNP200* (NM_014014.3: c.2042G>A, p.(Arg681His)), accounting for a diagnosis of autosomal dominant retinitis pigmentosa for this individual, with no pathogenic/likely pathogenic variants identified in-trans to the confirmed heterozygous deletion of *IDH3B* and *MKKS*. Of the 25 CNVs that enabled the confirmation or provisional confirmation of diagnosis, 23 confirmed autosomal recessive disorders and 2 confirmed autosomal dominant disorders (online supplemetary table S5). Twenty of these CNV events were confirmed in a heterozygous state, with 18 of the events suspected (n=8) or confirmed (n=10) to be in-trans to a heterozygous and proven/potentially pathogenic SNV or indel confirmed within the same gene (online supplementary table S5). Confirmation of in-trans variants included the encapsulation of an apparently homozygous SNV/indel by a heterozygous deletion event and/or familial segregation analysis. For example, a heterozygous whole gene deletion of *RPE65* (*NM_000329.2*) was identified for an individual originally described with a clearly pathogenic homozygous missense variant (*NM_000329.2:* c.1102T>C, p.(Tyr368His)). Subsequent familial segregation analysis confirmed these events to be paternally and maternally inherited, respectively. Five homozygous CNV events were confirmed to account for a molecular diagnosis for referred individuals, including four homozygous deletions (table 1) and a single duplication event confirmed as four copies of *EYS* exons 34–35 (*NM_001142800.1*).

**Table 1:** Confirmed CNVs impacting genes known as a cause of inherited retinal disease

Study ID	Gene	Zygosity	*hg19* coordinates of implicated exons	Exons (n)	HGVS cDNA	Classification
Deletions						
15010656	TRPM1	Het	chr15:31294020–31369129	26	NM_002420.5: c.(?_−1)_(*1_?)del	Likely pathogenic
14016924	PDE6B	Het	chr4:6 19 411–6 63 901	22	NM_000283.3: c.(?_−1)_(*1_?)del	Likely pathogenic
15000307	MERTK	Het	chr2:112702532–112786446	17	NM_006343.2: c.(482+1_483–1)_(3000+1_3001–1)del	Likely pathogenic
15010972*	PCDH15	Het	chr10:55826512–56424027	19	NM_001142763.1: c.(?_−1)_(2235+1_2236–1)del	Likely pathogenic
15012122	KIF11	Het	chr10:94389928–94413558	11	NM_004523.3: c.(1305+1_1306–1)_(*1_?)del	Likely pathogenic
15006709*	MERTK	Het	chr2:112656308–112733054	7	NM_006343.2: c.(?_−1)_(1144+1_1145–1)del	Likely pathogenic
084929	RPE65	Het	chr1:68895454–68915593	14	NM_000329.2: c.(?_−1)_(*1_?)del	Likely pathogenic
15005941	USH2A	Het	chr1:216405290–216465717	5	NM_206933.2: c.(1644+1_1645–1)_(2993+1_2994–1)del	Likely pathogenic
15005265	EYS	Het	chr6:65612001–65655812	4	NM_001142800.1: c.(2259+1_2260–1)_(2846+1_2847–1)del	Likely pathogenic
14015843	CRB1	Het	chr1:197390125–197397136	2	NM_201253.2: c.(1171+1_1172–1)_(2676+1_2677–1)del	Likely pathogenic
13011434*	EYS	Hom	chr6:64708964–64709081	1	NM_001142800.1: c.(6725+1_6726–1)_(6834+1_6835–1)del	Likely pathogenic
15005668*	CERKL	Het	chr2:182468559–182521738	2	NM_001030311.2: c.(?_−1)_(481+1_482–1)del	Likely pathogenic
15010867*	CNGB3	Het	chr8:87655974–87656919	2	NM_019098.4: c.(990+1_991–1)_(1178+1_1179–1)del	Likely pathogenic
15005008*	NMNAT1	Het	chr1:10035645–10035838	1	NM_001297778.1: c.(115+1_116–1)_(299+1_300–1)del	Likely pathogenic
12008422*	USH2A	Het	chr1:216172225–216173909	2	NM_206933.2: c.(6325+1_6326–1)_(6657+1_6658–1)del	Likely pathogenic
14017566	CERKL	Het	chr2:182521491–182521738	1	NM_001030311.2: c.(?_−1)_(238+1_239–1)del	Likely pathogenic
15001263*	USH2A	Het	chr1:216011328–216011450	1	NM_206933.2: c.(9258+1_9259–1)_(9371+1_9372–1)del	Likely pathogenic
15004859*	RPGRIP1	Het	chr14:21798403–21798551	1	NM_020366.3: c.(3099+1_3100–1)_(3238+1_3239–1)del	Likely pathogenic
13001147	EYS	Het	chr6:64791745–64791900	1	NM_001142800.1: c.(6424+1_6425–1)_(6571+1_6572–1)del	Likely pathogenic
13006640	LRP5	Het	chr11:68178900–68179093	1	NM_002335.2: c.(2318+1_2319–1)_(2503+1_2504–1)del	Likely pathogenic
14010419	CNGB1	Hom	chr16:57937722–57946903	4	NM_001297.4: c.(2304+1_2305–1)_(2794+1_2795–1)del	Likely pathogenic
12014502	CNGB1	Hom	chr16:57937722–57946903	4	NM_001297.4: c.(2304+1_2305–1)_(2794+1_2795–1)del	Likely pathogenic
14020104	MAK	Hom	chr6:10819114–10819178	1	NM_001242957.2: c.(101+1_102–1)_(156+1_157–1)del	Likely pathogenic
15010966	BBS2	Het	chr16:56544766–56545201	2	NM_031885.3: c.(345+1_346–1)_(534+1_535–1)del	Likely pathogenic
14017272	BBS4	Het	chr15:73015130–73017001	2	NM_033028.4:c.(405+1_406–1)_(587+1_588–1)del	Likely pathogenic
14021329	CDH3	Het	chr16:68721410–68725834	2	NM_001793.5:c.(1570+1_1571–1)_(2002+1_2003–1)del	Likely pathogenic
15010313	CLN3	Het	chr16:28497663–28497976	2	NM_001042432.1:c.(460+1_461–1)_(677+1_678–1)del	Likely pathogenic
14016318	GRM6	Het	chr5:178413126–178413759	1	NM_000843.3:c.(1500+1_1501)_(2124+1_2125–1)del	Likely pathogenic
13009597†	IDH3B	Het	chr20:2639084–10394167	17	NM_006899.4:c.(?_−1)_(*1_?)del	Likely pathogenic
	MKKS				NM_018848.3:c.(?_−1)_(*1_?)del	
14009753	NPHP1	Het	chr2:110881363–110962550	20	NM_000272.3:c.(?_−1)_(*1_?)del	Likely pathogenic
13013491	RGR	Het	chr10:86008662–86008804	1	NM_002921.3:c.(236+1_237–1)_(370+1_371–1)del	Uncertain significance
043844	FSCN2	Het	chr17:79502074–79502239	1	NM_001077182.2:c.(826+1_827–1)_(983+1_984–1)del	Uncertain significance
14020099	RP1L1	Het	chr8:10473951–10480716	2	NM_178857.5:c.(?_−1)_(751+1_752–1)del	Uncertain significance
Duplications						
10003406	USH2A	Het	chr1:215914713–215933190	4	NM_206933.2: c.(11048+1_11 049–1)_(11711+1_11 712–1)dup	Likely pathogenic
13018538‡	EYS	Hom	chr6:65016859–65016980	1	NM_001142800.1: c.(6078+1_6079–1)_(6191+1_6192–1)dup	Likely pathogenic
14001342	EYS	Het	chr6:64694272–64709081	2	NM_001142800.1: c.(6725+1_6726–1)_(7055+1_7056–1)dup	Uncertain significance
14017670‡	PRPF31	Hom	chr19:54621654–54628040	7	NM_015629.3: c.(?_−1)_(855+1_856–1)dup	Uncertain significance
15007281	BBS5	Het	chr2:170336059–170361097	12	NM_152384.2:c.(?_−1)_(*1_?)dup	Uncertain significance
15009450†	RP9 BBS9	Het	chr7:33134841–33185981	7	NM_203288.1:c.(?_−1)_(*1_?)dup NM_198428.2:c.(?_−1)_(112+1_113–1_?)dup	Uncertain significance
13009597†	ZNF513 C2orf71 EFEMP1 FAM161A	Het	chr2:27600408–62081181	23	NM_144631.5:c.(?_−1)_(*1_?)dup NM_001029883.2:c.(?_−1)_(*1_?)dup NM_001039348.2:c.(?_−1)_(*1_?)dup NM_001201542.1: c.(?_−1)_(*1_?)dup	Uncertain significance
14015751	NPHP1	Het	chr2:110881363–110962550	20	NM_000272.3:c.(?_−1)_(*1_?)dup	Likely benign
14018818	NPHP1	Het	chr2:110881363–110962550	20	NM_000272.3:c.(?_−1)_(*1_?)dup	Likely benign
15008560	NPHP1	Het	chr2:110881363–110962550	20	NM_000272.3:c.(?_−1)_(*1_?)dup	Likely benign
15010871	CYP4V2	Het	chr4:187112973–187131800	20	NM_207352.3:c.(?_−1)_(*1_?)dup	Likely benign

*CNV events reported previously in Ellingford *et al*,^14^ Ellingford *et al* 7 or Carss *et al* 18 through alternative techniques and analysis strategies.

†Four copies confirmed.

‡CNV event impacts multiple genes.

Het, heterozygous; hom, homozygous.

We confirmed that seven ‘likely pathogenic’ deletions were present in a carrier state, including two whole gene deletions, two deletions predicted to cause a frameshift and three inframe deletions. These events were all described in genes known as a cause of IRD or associated syndromic disorders that are inherited in an autosomal recessive manner, including *BBS2*, *BBS4*, *CDH3*, *CLN3*, *GRM6*, *NPHP1*, and a deletion spanning *IDH3B* and *MKKS* (table 1).

Duplications proved more complex for clinical interpretation, and based on current evidence most of the identified duplications were classified as ‘uncertain significance’ (45%, n=5) or to be ‘likely benign’ (36%, n=4).

In four individuals, we identified heterozygous CNV events in genes known as a cause of autosomal dominant Mendelian disorders that were not determined to be a cause of disease for the referred individual (table 1). These included a three-exon deletion in *RP1L1* (*NM_178857.5*), a single-exon deletion in *FSCN2* (*NM_001077182.2*), a single-exon deletion in *RGR* (*NM_002921.3*) and a duplication event impacting *RP9* (*NM_203288.1*) and *BBS9* (*NM_198428.2*). Of note, we also identified four copies of *PRPF31* exons 2–8 (*NM_015629.3*) in an additional individual. Based on current evidence, the *PRPF31* duplication was classified as ‘uncertain significance’ (online supplementary case study), although we expect future investigations to assist with the interpretation of this variant.

### Population and in-house frequencies of identified CNV events

To assist with clinical interpretation, the frequency of confirmed CNV events was determined through comparison to two independently acquired cohorts of WGS data sets generated for individuals with a clinical indication of IRD (605 through the NIHR BioResource Rare Diseases project using Illumina sequencing, and 77 through Complete Genomics sequencing). Of the 44 confirmed CNV events reported in this study, 25 (57%) were found to have an overlap with events identified through WGS. This analysis was restricted to events identified through WGS, which overlapped at least 50% of the event identified through gene panel NGS. Three of these samples with identified CNV events were also included in the WGS cohorts (two from Illumina sequencing and one from Complete Genomics sequencing), enabling an assessment of the relative advantages for detecting CNVs through WGS in comparison to gene panel NGS (online supplementary figures S4, S5 and table 6) (should be table S6). Seven events were identified to have an overlap with more than one individual within the WGS cohorts (table 2). Of note, a confirmed duplication of *RP9/BBS9* was identified in four unrelated WGS samples through Illumina sequencing (online supplementary figure S5). This information, in complement to other confirmed SNVs/indels for these individuals, permitted the classification of this duplication event as ‘uncertain significance’ and unlikely to account for the individual’s molecular diagnosis. Similarly, whole gene duplication events of *NPHP1* and *CYP4V2* were identified in multiple unrelated individuals across the cohorts, and the absence of a second disease-causing mutation in these genes in all reported cases suggests they may represent benign variation. Future investigations into the pathogenicity of whole gene duplication events will assist with interpretation and will provide greater clarity in clinical reporting. These investigations may consist of WGS and/or long-read NGS to better characterise the location and phase of duplications, and RNA-seq experiments to assess the effect of duplications on gene expression.

**Table 2:** CNVs identified in more than one unrelated individual

CNV event	CNV type	Individuals (n)			
		Total	Gene panel NGS, n=550	WGS (Illumina) n=605	WGS (Complete Genomics) n=77
CLN3 ex8-9	Del	6	1	5	0
MERTK ex1-7	Del	3	1	1	1
NPHP1 (whole gene)	Del/Dup	7/10	1/3	5/6	1/1
RP9/BBS9	Dup	5	1	4	0
CNGB3 ex9-10	Del	4	1	3*	0
CYP4V2 (whole gene)	Dup	5	1	4	0

*Indicated, but unconfirmed, as *CNGB3* ex7-10 for two individuals, and *CNGB3* ex8-10 for one individual using the Canvas read-depth algorithm.

Del, deletions; dup, duplications; NGS, next-generation sequencing; WGS, whole genome sequencing.

## Discussion

A variety of techniques exist for the identification of genomic CNVs, including MLPA, Q-PCR, genome-wide and customised array CGH, and low-coverage genome-wide sequencing.^11^ The detection of CNVs from high-coverage NGS data provides the unique opportunity for the simultaneous analysis of novel disease-causing SNVs and small indels, a strategy that has proved extremely successful for the diagnosis of IRD.^9^ While a number of informatics techniques exist for the identification of CNVs from NGS data sets,^28^ gene panel NGS approaches are limited by the types of CNV detection algorithms which can be routinely applied. Here, we describe an implemented informatics strategy using read-depth algorithms for the identification of CNVs from gene panel NGS data sets for 550 individuals with IRD. Through these strategies, we have confirmed 33 deletions and 11 duplications (table 1), determining these findings to contribute to the molecular diagnosis or provisional molecular diagnosis of IRD for 25 individuals (online supplementary table S5).

This study provides the largest cohort, to date, for the assessment of the relative frequency of CNVs as a cause of IRD from targeted NGS data sets. Our group and others have estimated the contribution of CNVs in IRDs from smaller cohorts of individuals, including high-resolution array CGH approaches (3.5%, n=57),^16^ gene panel NGS (3.1%, n=126; 1.1%, n=89; 6.4%, n=47),^19 29 30^ WES (10%, n=60)^17^ and WGS (10.9%, n=46; 12.5%, n=16).^7 31^ Here, we show that CNVs contribute to a molecular diagnosis of IRD in 4.5% of cases, and are found without contribution to a molecular diagnosis in a further 3.3% of cases. Altogether, we estimate that a CNV is present within IRD genes in at least 1 in 13 individuals presenting with IRD, and thereby provides a significant and essential component of the diagnostic assessment.

The incorporation of read-depth CNV detection algorithms into gene panel NGS diagnostic services for IRD provides a realistic and cost-effective opportunity for widespread incorporation of CNV analysis. However, false-negative assessments, false-positive discoveries, complexity with clinical interpretation and the size of events that can be detected all provide significant limitations to this approach.^32^ To overcome these challenges in this study, we compared the results from ExomeDepth with two other publicly available CNV detection algorithms with the capability to detect single-exon CNV events (CoNVex^22^ and CoNVaDING^12^) and used distinct strategies for CNV filtering to reduce the number of false-positive events analysed (figure 1). These filtering approaches provided a PPV of 79% (44/56) and enabled the confirmation of events with a range of confidence scores calculated by the ExomeDepth algorithm (min=6.7, max=424), including 11 single-exon deletions and one single-exon duplication. Furthermore, we assessed two key quality assurance parameters previously identified as key determinants of false-negative assessments through ExomeDepth: insufficient coverage and intersample variability.^14^ We identified that 99.2% of surveyed exons had appropriate sequencing coverage for CNV surveillance in tested samples and that 99% of exons were consistent with a 98.2% accuracy of ExomeDepth in comparison to 1000 previously reported simulated single-exon deletion events.^14^ Importantly, the frequency of CNVs reported for this cohort are concordant with a recent study that interrogated rare variants in 224 IRD-associated genes from WGS data sets for 605 individuals with IRD,^18^ and these data provide additional support for the sensitivity of the methodologies applied to gene panel NGS data sets in this study.

We have described CNVs in 36 different genes. The genes most frequently identified with CNVs were *EYS* (n=5), *USH2A* (n=4) and *NPHP1* (n=4) (table 1). These data are in accordance with recent findings that have identified factors underpinning susceptibility of IRD genes to CNVs.^33^ Microhomology-mediated DNA repair mechanisms (eg, microhomology-mediated break-induced replication) have been proposed as a major contributor to the genesis of non-recurrent CNVs.^33 34^ Our data sets precluded a comprehensive assessment of CNV mechanisms. However, it is notable that we have observed small stretches of microhomology between proximal and distal genomic sequences at breakpoints for non-recurrent CNVs (online supplementary table S6). We have also identified several instances of a recurrent duplication and a recurrent deletion of the complete coding region of *NPHP1* (*NM_000272.3*), which are expected to have arisen through non-allelic homologous recombination between segmental duplications flanking *NPHP1*.^35^ The deletion of *NPHP1* has been frequently reported as a cause of autosomal recessive juvenile nephronophthisis and Senior-Loken syndrome. The emergence of long-read NGS techniques to study CNVs will likely assist in the comprehensive characterisation of structural variant breakpoints, the elucidation of CNV genesis mechanisms, and the existence of ancestral and susceptibility haplotypes for CNVs that impact IRD genes.

In total we confirmed 44 CNV events through the described informatics strategies (figure 1), including 12 whole gene events, 6 events removing or duplicating the canonical start or end codon, and 26 intragenic events. These strategies validated the presence of 28% and 16% of the deletions and duplications originally identified by ExomeDepth, respectively (figure 1). While these data suggest that IRD genes are more susceptible to deletion than duplication, our observations may be a limitation of the approaches applied, as NGS read-depth CNV detection software has been shown to be less sensitive for small duplication events.^36^ Duplications also proved more challenging for clinical interpretation as we were unable to determine phase of apparently homozygous events or confirm the genomic location of duplicated sequences. Both of these identified challenges may be overcome by the application of split-read and discordant read-pair algorithms to WGS data sets.^28^ A duplication identified in *PRPF31*, confirmed to be two extra copies of exons 2–8, proved particularly problematic for clinical interpretation (online supplementary case study). Recently, Ayuso *et al* identified that a heterozygous duplication in *PRPF31*, encompassing exons 2–5, significantly reduced gene expression of *PRPF31* and underpinned clinical presentation of retinitis pigmentosa.^37^ These results are consistent with the haploinsufficient pathogenic mechanism of mutations in *PRPF31* and other pre-mRNA splicing factor genes.^38^ However, mutations in *PRPF31* are often reported with incomplete penetrance,^38^ and the patient identified with this duplication in our cohort also carried a homozygous variant in another gene surveyed through gene panel NGS that could account for their molecular diagnosis of IRD  (online supplementary case study). Future assessments of the location of duplicated sequences and their effect on *PRPF31* gene expression will assist with clinical interpretation and will be of great interest.

Interestingly, we also identified a number of genes that were absent from CNVs, including *ABCA4*, one of the most prevalent causes of IRD and a gene commonly identified to be in a carrier state in tested individuals. While it is possible that sequencing data generated for *ABCA4* have characteristics that reduce the accuracy of the read-depth CNV detection techniques described here, none of the three applied algorithms identified deletions or duplications disrupting or encapsulating *ABCA4*, the sequencing profile is consistent with accurate surveillance of CNVs (onlinesupplementary table S7), and these findings are consistent with the absence and rare occurrence of CNVs in *ABCA4* in studies using WGS and array CGH for CNV interrogation.^18 39 40^

Taken together, we demonstrate that CNVs provide a significant contribution towards the onset of IRD. We show that read-depth algorithms applied to gene panel NGS data sets generated for individuals with IRD can identify deletion and duplication events ranging from single exons to multigene events, and provide compelling evidence for the routine incorporation of CNV analysis as a first-tier diagnostic test for individuals with IRD.
