# Supplementary material for: Assessment of the incorporation of CNV surveillance into gene panel next-generation sequencing testing for inherited retinal diseases
Source: J Med Genet. 2017 Oct 26;55(2):114–21. doi: 10.1136/jmedgenet-2017-104791 (PMC5800348; doi:10.1136/jmedgenet-2017-104791)
Supplement: Supplementary file 1 [file jmedgenet-2017-104791supp001.docx]

## Supplemental Results

**Informatics strategies to reduce false positives**

In total, ExomeDepth identified 187 potential CNVs, with ≥1 CNV identified in 121 individuals. In order to reduce the number of false positive events analysed in a clinical context, we used three distinct strategies for CNV filtering. In combination, these analyses identified 56 CNV events from 52 individuals for further confirmation and clinical analysis (Figure 1).

**Overlap with other copy number variant detection algorithms**

CNV calling was performed using two other publically available CNV detection software tools (CoNVex and CoNVaDING), using the same 4 groups of reference sets matched by gender and enrichment kit that were parsed to ExomeDepth. We found that 105 CNV events (56% of original 187; 77 deletions, 28 duplications) identified by ExomeDepth were overlapped by calls from CoNVex (*n*=12, 7 deletions, 5 duplications), CoNVaDING (*n*=54, 34 deletions, 20 duplications) or both (*n*=39, 36 deletions, 3 duplications). In comparison to ExomeDepth, CoNVex identified more potential CNV events (Figure S1, *n*=921), and more individuals with at least 1 CNV (Figure S1, *n*=293). In contrast, CONVaDING identified more potential CNV events (Figure S1, *n*=518), but less individuals had at least 1 CNV than observed with ExomeDepth (Figure S1, *n*=72). CONVaDING had the additional limitation that apparently continuous CNV events were fragmented into 2 or more events in 22 samples. On average, ExomeDepth identified 0.21 deletions and 0.13 duplications per individual, whereas CoNVex and CONVaDING identified 0.97 and 0.71 deletions, and 0.71 and 0.36 duplications, respectively.

**Overlap between mutually exclusive reference sets**

We also performed CNV calling for tested samples using ExomeDepth with 3 mutually exclusive reference sets, each comprised of 30 unrelated individuals referred for diagnostic genomic testing. We found 118 CNV events (63% of original 187; 84 deletions and 34 duplications) that were identified in comparison to all 3 reference sets. This included 80 CNV events (63 deletions and 17 duplications) that were also identified by CoNVex, CONVaDING, or both, five of which encapsulated the complete enriched region of chrX and were discarded as gender mismatches. Eight of the CNV events identified through this consensus approach to running ExomeDepth were not present in the original 187 events. None of these events overlapped with CNV events from other algorithms, and all were disregarded before visual inspection.

**Visual inspection**

All 75 CNV events that shared an overlap between the ExomeDepth consensus approach and other algorithms were visualized using a graphical tool. Each event was assessed for the consistency of calculated read ratios across all exons within implicated genes, the extent of variation within the selected reference samples for each exon, the nature of the exon CNV status across the cohort, and the continuity of abnormal CNV exons within the gene. As a result of these analyses, 19 events were excluded from further analysis and 56 events were selected for confirmation through orthogonal techniques (Figure 1).

**Copy number variant confirmations**

Of the 44 confirmed events, 11 were confirmed through MLPA techniques, using kits available from MRC-Holland (<http://www.mrc-holland.com/>). These included three *USH2A* events (MLPA kits: SALSA MLPA P361 USH2A mix-1 probemix, SALSA MLPA P362 USH2A mix-1 probemix), five *EYS* events (MLPA kit: SALSA MLPA P328 EYS probemix), a *CRB1* deletion (MLPA kit: SALSA MLPA P221 LCA mix-1 probemix), a *RPE65* deletion (MLPA kit: SALSA MLPA P221 LCA mix-1 probemix) and a *PRPF31* duplication (MLPA kit: SALSA MLPA P235 Retinitis probemix). The remaining events were confirmed through quantitative fluorescence PCR or droplet digital PCR approaches. These full methodologies have been reported previously.^14^ Briefly, both techniques allow a quantitative assessment, through optical sensing, of the relative level of amplification for an investigated region (targeted through customized PCR primers) in comparison to defined control regions in the same sample. Primer sequences used for variant confirmation will be made available upon request.

Thirteen events that were excluded from consideration through comparison to CoNVex and CoNVaDING were also investigated through a droplet digital PCR technique. One of the thirteen events was confirmed as a single likely benign duplication in *NPHP1* (*14016366*; NM_000272.3:c.(?_-1)_(*1_?)dup), but the remaining 12 events were determined to have no deviation from the copy number status of control samples. This analysis was restricted to 13 CNV events that were selected from those present in the overlap between mutually exclusive reference sets (n=32, 13 duplications, 19 deletions). Of these 32 events, 21 (65%) are accounted for by 2 reoccurring CNV events (13 heterozygous deletions in *BBS9* and 9 duplications in *RP9*, 10 of which were investigated through ddPCR) and 5 (16%) are present in a single sample which consistently matches poorly to presented reference sets.

## Supplemental Tables

**Table S1. Specified transcripts for genes causing inherited retinal disease that were enriched and analysed through gene panel NGS.**

| HGNC | Transcript | HGNC | Transcript | HGNC | Transcript |
| --- | --- | --- | --- | --- | --- |
| *ABCA4* | NM_000350.2 | *PRPF31* | NM_015629.3 | *IMPG2* | NM_016247.3 |
| *ADAM9* | NM_003816.2 | *PRPF6* | NM_012469.3 | *KCNV2* | NM_133497.3 |
| *AIPL1* | NM_014336.3 | *PRPF8* | NM_006445.3 | *KLHL7* | NM_001031710.2 |
| *ARL6* | NM_032146.3 | *PRPH2* | NM_000322.4 | *IMPG2* | NM_016247.3 |
| *BBS1* | NM_024649.4 | *RAX2* | NM_032753.3 | *KCNV2* | NM_133497.3 |
| *BBS10* | NM_024685.3 | *RBP3* | NM_002900.2 | *KLHL7* | NM_001031710.2 |
| *BBS12* | NM_001178007.1 | *RD3* | NM_183059.2 | *LCA5* | NM_181714.3 |
| *BBS2* | NM_031885.3 | *RDH12* | NM_152443.2 | *LRAT* | NM_004744.3 |
| *BBS4* | NM_033028.3 | *RDH5* | NM_001199771.1 | *LRP5* | NM_002335.2 |
| *BBS5* | NM_152384.2 | *RGR* | NM_002921.3 | *MERTK* | NM_006343.2 |
| *BBS7* | NM_176824.2 | *RGS9* | NM_001165933.1 | *MKKS* | NM_018848.2 |
| *BBS7* | NM_018190.3 | *RGS9* | NM_003835.3 | *MKS1* | NM_001165927.1 |
| *BBS9* | NM_198428.2 | *RHO* | NM_000539.3 | *MKS1* | NM_017777.3 |
| *BEST1* | NM_004183.3 | *RIMS1* | NM_014989.4 | *MYO7A* | NM_000260.3 |
| *C1QTNF5* | NM_015645.3 | *RIMS1* | NM_001168407.1 | *NDP* | NM_000266.3 |
| *C2orf71* | NM_001029883.1 | *CRB1* | NM_201253.2 | *RIMS1* | NM_001168410.1 |
| *CA4* | NM_000717.3 | *CRX* | NM_000554.4 | *RLBP1* | NM_000326.4 |
| *CACNA2D4* | NM_172364.4 | *DFNB31* | NM_015404.3 | *ROM1* | NM_000327.3 |
| *CDH23* | NM_022124.5 | *DHDDS* | NM_024887.2 | *RP1* | NM_006269.1 |
| *CDHR1* | NM_001171971.1 | *EFEMP1* | NM_001039348.2 | *RP1L1*** | NM_178857.5 |
| *CDHR1* | NM_033100.2 | *ELOVL4* | NM_022726.3 | *RP2* | NM_006915.2 |
| *CEP290** | NM_025114.3 | *EYS* | NM_001142800.1 | *RP9* | NM_203288.1 |
| *CERKL* | NM_001030311.2 | *FAM161A* | NM_001201543.1 | *RPE65* | NM_000329.2 |
| *CHM* | NM_000390.2 | *FSCN2* | NM_001077182.2 | *RPGR**** | NM_001034853.1 |
| *CLRN1* | NM_052995.2 | *FZD4* | NM_012193.3 | *RPGRIP1* | NM_020366.3 |
| *CLRN1* | NM_001195794.1 | *GNAT2* | NM_005272.3 | *RS1* | NM_000330.3 |
| *CNGA1* | NM_001142564.1 | *GPR98* | NM_032119.3 | *SAG* | NM_000541.4 |
| *CNGA3* | NM_001298.2 | *GUCA1A* | NM_000409.3 | *SEMA4A* | NM_022367.3 |
| *CNGB1* | NM_001297.4 | *GUCA1B* | NM_002098.5 | *SNRNP200* | NM_014014.4 |
| *CNGB3* | NM_019098.4 | *GUCY2D* | NM_000180.3 | *SPATA7* | NM_018418.4 |
| *NR2E3* | NM_014249.2 | *IDH3B* | NM_006899.2 | *TEAD1* | NM_021961.5 |
| *NRL* | NM_006177.3 | *IDH3B* | NM_174855.1 | *TIMP3* | NM_000362.4 |
| *OTX2* | NM_021728.2 | *IMPDH1* | NM_000883.3 | *TOPORS* | NM_005802.4 |
| *PCDH15* | NM_001142763.1 | *IMPG2* | NM_016247.3 | *TRIM32* | NM_012210.3 |
| *PCDH15* | NM_001142769.1 | *KCNV2* | NM_133497.3 | *TTC8* | NM_144596.2 |
| *PCDH15* | NM_001142771.1 | *KLHL7* | NM_001031710.2 | *TULP1* | NM_003322.3 |
| *PCDH15* | NM_001142770.1 | *LCA5* | NM_181714.3 | *UNC119* | NM_005148.3 |
| *PDE6A* | NM_000440.2 | *LRAT* | NM_004744.3 | *UNC119* | NM_054035.2 |
| *PDE6B* | NM_000283.3 | *LRP5* | NM_002335.2 | *USH1C* | NM_005709.3 |
| *PDE6C* | NM_006204.3 | *MERTK* | NM_006343.2 | *USH1C* | NM_153676.3 |
| *PDE6G* | NM_002602.3 | *MKKS* | NM_018848.2 | *USH1G* | NM_173477.2 |
| *PITPNM3* | NM_031220.3 | *MKS1* | NM_001165927.1 | *USH2A* | NM_206933.2 |
| *PRCD* | NM_001077620.2 | *MKS1* | NM_017777.3 | *ZNF513* | NM_144631.5 |
| *PROM1* | NM_006017.2 | *MYO7A* | NM_000260.3 |  |  |
| *PRPF3* | NM_004698.2 | *NDP* | NM_000266.3 |  |  |
|  |  |  |  |  |  |

1. RDv2 – 105 genes

Testing of the common intron 26 mutation c.2991+1655A>G in CEP290 is included in this analysis. ** Analysis of the coding region of exon 4 of the RP1L1 gene is not included. *** Analysis of the coding region of the final exon (*orf15*) of *RPGR* it is not included.

1. RDv3 – 180 genes

| HGNC | Transcript | HGNC | Transcript | HGNC | Transcript |
| --- | --- | --- | --- | --- | --- |
| *ABCA4* | NM_000350 | *GNAT1* | NM_000172 | *PEX7* | NM_000288 |
| *ABHD12* | NM_001042472 | *GNAT2* | NM_005272 | *PHYH* | NM_006214 |
| *ACBD5* | NM_145698 | *GNPTG* | NM_032520 | *PITPNM3* | NM_031220 |
| *ADAM9* | NM_003816 | *GPR125* | NM_145290 | *PLA2G5* | NM_000929 |
| *ADAMTS18* | NM_199355 | *GPR179* | NM_001004334 | *PRCD* | NM_001077620 |
| *AHI1* | NM_001134832; NM_017651 | *GPR98* | NM_032119 | *PROM1* | NM_006017 |
| *AIPL1* | NM_014336 | *GRK1* | NM_002929 | *PRPF3* | NM_004698 |
| *ALMS1* | NM_015120 | *GRM6* | NM_000843 | *PRPF31* | NM_015629 |
| *ARL2BP* | NM_012106 | *GUCA1A* | NM_000409 | *PRPF4* | NM_004697 |
| *ARL6* | NM_032146 | *GUCA1B* | NM_002098 | *PRPF6* | NM_012469 |
| *BBIP1* | NM_001195306 | *GUCY2D* | NM_000180 | *PRPF8* | NM_006445 |
| *BBS1* | NM_024649 | *HARS* | NM_002109 | *PRPH2* | NM_000322 |
| *BBS10* | NM_024685 | *HMX1* | NM_018942 | *RAB28* | NM_001017979 |
| *BBS12* | NM_001178007 | *IDH3B* | NM_006899; NM_174855 | *RAX2* | NM_032753 |
| *BBS2* | NM_031885 | *IFT140* | NM_014714 | *RBP3* | NM_002900 |
| *BBS4* | NM_033028 | *IMPDH1* | NM_000883 | *RBP4* | NM_006744 |
| *BBS5* | NM_152384 | *IMPG1* | NM_001563 | *RD3* | NM_183059 |
| *BBS7* | NM_176824 | *IMPG2* | NM_016247 | *RDH12* | NM_152443 |
| *BBS9* | NM_198428 | *INPP5E* | NM_019892 | *RDH5* | NM_001199771 |
| *BEST1* | NM_004183 | *INVS* | NM_014425 | *RGR* | NM_002921 |
| *C1QTNF5* | NM_015645 | *IQCB1* | NM_001023570 | *RGS9* | NM_001165933; NM_003835 |
| *C21orf2* | NM_004928 | *ITM2B* | NM_021999 | *RHO* | NM_000539 |
| *C2orf71* | NM_001029883 | *KCNJ13* | NM_002242 | *RIMS1* | NM_001168407; NM_001168410; NM_014989 |
| *C8orf37* | NM_177965 | *KCNV2* | NM_133497 | *RLBP1* | NM_000326 |
| *CA4* | NM_000717 | *KIAA1549* | NM_001164665; NM_020910 | *ROM1* | NM_000327 |
| *CABP4* | NM_145200 | *KIF11* | NM_004523 | *RP1* | NM_006269 |
| *CACNA1F* | NM_005183 | *KLHL7* | NM_001031710 | *RP1L1* | NM_178857 |
| *CACNA2D4* | NM_172364 | *LCA5* | NM_181714 | *RP2* | NM_006915 |
| *CAPN5* | NM_004055 | *LRAT* | NM_004744 | *RP9* | NM_203288 |
| *CC2D2A* | NM_001080522 | *LRIT3* | NM_198506 | *RPE65* | NM_000329 |
| *CDH23* | NM_022124 | *LRP5* | NM_002335 | *RPGR* | NM_001034853 |
| *CDH3* | NM_001793 | *LZTFL1* | NM_020347 | *RPGRIP1* | NM_020366 |
| *CDHR1* | NM_001171971; NM_033100 | *MAK* | NM_001242957 | *RPGRIP1L* | NM_015272 |
| *CEP164* | NM_014956 | *MERTK* | NM_006343 | *RS1* | NM_000330 |
| *CEP290* | NM_025114 | *MFRP* | NM_031433 | *SAG* | NM_000541 |
| *CERKL* | NM_001030311 | *MKKS* | NM_018848 | *SDCCAG8* | NM_006642 |
| *CHM* | NM_000390 | *MKS1* | NM_001165927; NM_017777 | *SEMA4A* | NM_022367 |
| *CIB2* | NM_006383 | *MVK* | NM_000431 | *SLC24A1* | NM_001254740; NM_004727 |
| *CLN3* | NM_000086 | *MYO7A* | NM_000260 | *SNRNP200* | NM_014014 |
| *CLRN1* | NM_001195794; NM_052995 | *NDP* | NM_000266 | *SPATA7* | NM_018418 |
| *CNGA1* | NM_001142564 | *NEK2* | NM_002497 | *TEAD1* | NM_021961 |
| *CNGA3* | NM_001298 | *NMNAT1* | NM_022787 | *TIMP3* | NM_000362 |
| *CNGB1* | NM_001297 | *NPHP1* | NM_000272 | *TMEM237* | NM_001044385 |
| *CNGB3* | NM_019098 | *NPHP3* | NM_153240 | *TOPORS* | NM_005802 |
| *CNNM4* | NM_020184 | *NPHP4* | NM_015102 | *TRIM32* | NM_012210 |
| *CRB1* | NM_201253 | *NR2E3* | NM_014249 | *TRPM1* | NM_002420 |
| *CRX* | NM_000554 | *NRL* | NM_006177 | *TSPAN12* | NM_012338 |
| *CSPP1* | NM_024790 | *NYX* | NM_022567 | *TTC8* | NM_144596 |
| *CYP4V2* | NM_207352 | *OAT* | NM_000274 | *TUB* | NM_177972 |
| *DFNB31* | NM_015404 | *OFD1* | NM_003611 | *TULP1* | NM_003322 |
| *DHDDS* | NM_024887 | *OTX2* | NM_021728 | *UNC119* | NM_005148; NM_054035 |
| *DTHD1* | NM_001136536; NM_001170700 | *PANK2* | NM_153638 | *USH1C* | NM_005709; NM_153676 |
| *EFEMP1* | NM_001039348 | *PCDH15* | NM_001142763; NM_001142769; NM_001142770; NM_001142771 | *USH1G* | NM_173477 |
| *ELOVL4* | NM_022726 | *PCYT1A* | NM_005017 | *USH2A* | NM_206933 |
| *EMC1* | NM_015047 | *PDE6A* | NM_000440 | *VCAN* | NM_004385 |
| *EYS* | NM_001142800 | *PDE6B* | NM_000283 | *VPS13B* | NM_017890 |
| *FAM161A* | NM_001201543 | *PDE6C* | NM_006204 | *WDPCP* | NM_015910 |
| *FLVCR1* | NM_014053 | *PDE6G* | NM_002602 | *WDR19* | NM_025132 |
| *FSCN2* | NM_001077182 | *PEX1* | NM_000466 | *ZNF423* | NM_015069 |
| *FZD4* | NM_012193 | *PEX2* | NM_000318 | *ZNF513* | NM_144631 |

Testing of the following intronic mutations are also included in the RDv3 analysis: *ABCA4* c.4539+2001G>A, c.4539+2028C>T, c.5196+1056A>G, c.5196+1137G>A, c.5196+1216C>A, c.5461-10T>C; *CEP290* c.2991+1655A>G; *OFD1* c.935+706A>G; *USH2A* c.7595-2144A>G

**Table S2. Sample numbers matched by gender and enrichment kit.** Numbers of individuals reflect the numbers of BAM files presented to CNV detection algorithms using a standalone approach.

|  | RDv2 (105 genes) | RDv3 (180 genes) |
| --- | --- | --- |
| Total | **197** | **353** |
| Females | 93 | 168 |
| Males | 104 | 185 |

|  | Total | Males (*n*=289) | Females (*n*=261) |
| --- | --- | --- | --- |
| Total | 187 (0.34) | 114 (0.39) | 73 (0.28) |
| del | 117 (0.21) | 77 (0.27) | 40 (0.15) |
| dup | 70 (0.13) | 37 (0.13) | 33 (0.13) |
| RDv2 (105 genes) | 55 (0.27) | 31 (0.30) | 24 (0.26) |
| del | 35 (0.17) | 23 (0.22) | 12 (0.13) |
| dup | 20 (0.10) | 8 (0.08) | 12 (0.13) |
| RDv3 (176 genes) | 132 (0.37) | 83 (0.45) | 49 (0.29) |
| del | 82 (0.23) | 54 (0.29) | 28 (0.17) |
| dup | 50 (0.14) | 29 (0.16) | 21 (0.13) |

**Table S3. Numbers of copy number variants identified through ExomeDepth for 550 individuals with inherited retinal disease.**

The average number of copy number variants identified per individual are indicated in parentheses. The numbers of individuals used for each gene panel to calculate these statistics are indicated in Table S2.

**Table S4. The number of exons determined to have poor coverage through gene panel next-generation sequencing.**

| panel | % of individuals in cohort with poor coverage | | | | |
| --- | --- | --- | --- | --- | --- |
|  | **>40%** | **20-40%** | **5-20%** | **0.01-5%** | **0%** |
| *low coverage (<50x)* |  |  |  |  |  |
| v2 (105 genes) | 8 | 0 | 10 | 29 | 1543 |
| v3 (180 genes) | 0 | 0 | 0 | 45 | 2659 |
| *no coverage* |  |  |  |  |  |
| v2 (105 genes) | 2 | 2 | 1 | 4 | 1581 |
| v3 (180 genes) | 0 | 0 | 0 | 1 | 2703 |

| Patient ID | Expected inheritance pattern | Referred clinical indication of IRD | Gene | Allele 1 | Allele 2 |
| --- | --- | --- | --- | --- | --- |
| 14017566* | ar | RCD/RP | *CERKL* | c.238+2T>C | exon 1 del |
| 15005668 | ar | RCD/RP | *CERKL* | c.1381C>T p.(Arg461Ter) | exon 2 del |
| 12014502 | ar | RD | *CNGB1* | exons 25-27 del | exons 25-27 del |
| 14010419 | ar | RD | *CNGB1* | exons 25-27 del | exons 25-27 del |
| 15010867** | ar | BCM | *CNGB3* | c.990+1G>T | exon 9 del |
| 14015843 | ar | RD | *CRB1* | c.3121A>G p.(Met1041Val) | exons 6-7 del |
| 13011434 | ar | RCD/RP | *EYS* | exon 34 del | exon 34 del |
| 13018538 | ar | RCD/RP | *EYS* | exon 30 dup | exon 30 dup |
| 13001147 | ar | RCD/RP | *EYS* | c.9061G>C p.(Ala3021Pro) | exon 32 del |
| 15005265 | ar | RCD/RP | *EYS* | c.8054G>A p.(Gly2685Glu) | exons 15-18 del |
| 15012122 | ad | RD | *KIF11* | 11 exon del | - |
| 13006640 | ad | FEVR | *LRP5* | 1 exon del | - |
| 14020104 | ar | RCD/RP | *MAK* | exon 2 del | exon 2 del |
| 15000307 | ar | severe RCD/RP | *MERTK* | c.263C>T p.(Ser88Leu) | exons 3-19 del |
| 15006709* | ar | severe RCD/RP | *MERTK* | c.263C>T p.(Ser88Leu) | exon 7 del |
| 15005008** | ar | eoRD/LCA | *NMNAT1* | c.634G>A p.(Val212Met) | exon 3 del |
| 15010972 | ar | Usher | *PCDH15* | c.2639C>T p.(Ser880Leu) | exon 2 del |
| 14016924* | ar | RCD/RP | *PDE6B* | c.1697C>T p.(Ala566Val) | whole gene del |
| 084929** | ar | RD | *RPE65* | c.1102T>C p.(Tyr368His) | exons 1-14 del |
| 15004859** | ar | eoRD/LCA | *RPGRIP1* | c.2079C>G p.(Tyr693Ter) | exon 19 del |
| 15010656* | ar | CSNB | *TRPM1* | c.3004A>T p.(Ile1002Phe) | exons 2-27 del |
| 12008422* | ar | Usher | *USH2A* | c.6446C>A p.(Pro2146Gln) | exons 33-34 del |
| 10003406 | ar | Usher | *USH2A* | c.5776+1G>A | exons 57-60 dup |
| 15001263 | ar | RCD/RP | *USH2A* | c.2802T>G p.(Cys934Trp) | exon 47 del |
| 15005941** | ar | RCD/RP | *USH2A* | c.9974G>A p.(Gly3325Glu) | exons 10-14 del |

**Table S5. Disease-causing alleles identified through routine gene panel NGS testing and CNV surveillance.**

RCD/RP, rod-cone dystrophy or retinitis pigmentosa; RD, retinal dystrophy; BCM, blue cone monochromatism; FEVR, familial exudative vitreo-retinopathy; eoRD/LCA, early onset retinal dystrophy or Leber congenital amaurosis; Usher, Usher syndrome; CSNB, congenital stationary night blindness. *****originally identified as hom, deletion encapsulates it. **proven *in-trans* through segregation testing.

**Table S6. Microhomology detected for CNV breakpoints identified at single nucleotide resolution for three individuals with whole genome sequencing.**

| CNV event | Breakpoints (*hg19*) | Microhomology |
| --- | --- | --- |
| *USH2A* ex47 del | chr1:216,009,683-216,011,948 | TTGTTC |
| *USH2A* ex33-34 del | chr1:216,177,486-216,167,537 | TGTCT |
| *EYS* ex34 del | chr6:64,708,420-64,712,049 | A |

**Table S7. Quality assurance parameters for *ABCA4* exons surveyed for copy number variants.**

Reported statistics: mean(min-max; SD). *17 samples were excluded from the analysis of inter-sample variation due to the selection of only a single reference sample through ExomeDepth.

| metric | *n* exons surveyed | observation |
| --- | --- | --- |
| insufficient coverage  (*n* exons with nucleotides <50x) | 29,618 | 0 (0-0; 0) |
| inter-sample variation  (rpkmCV, %) | 28,690* | 5.29 (0.01-37.19; 2.45) |

## Supplemental Figures


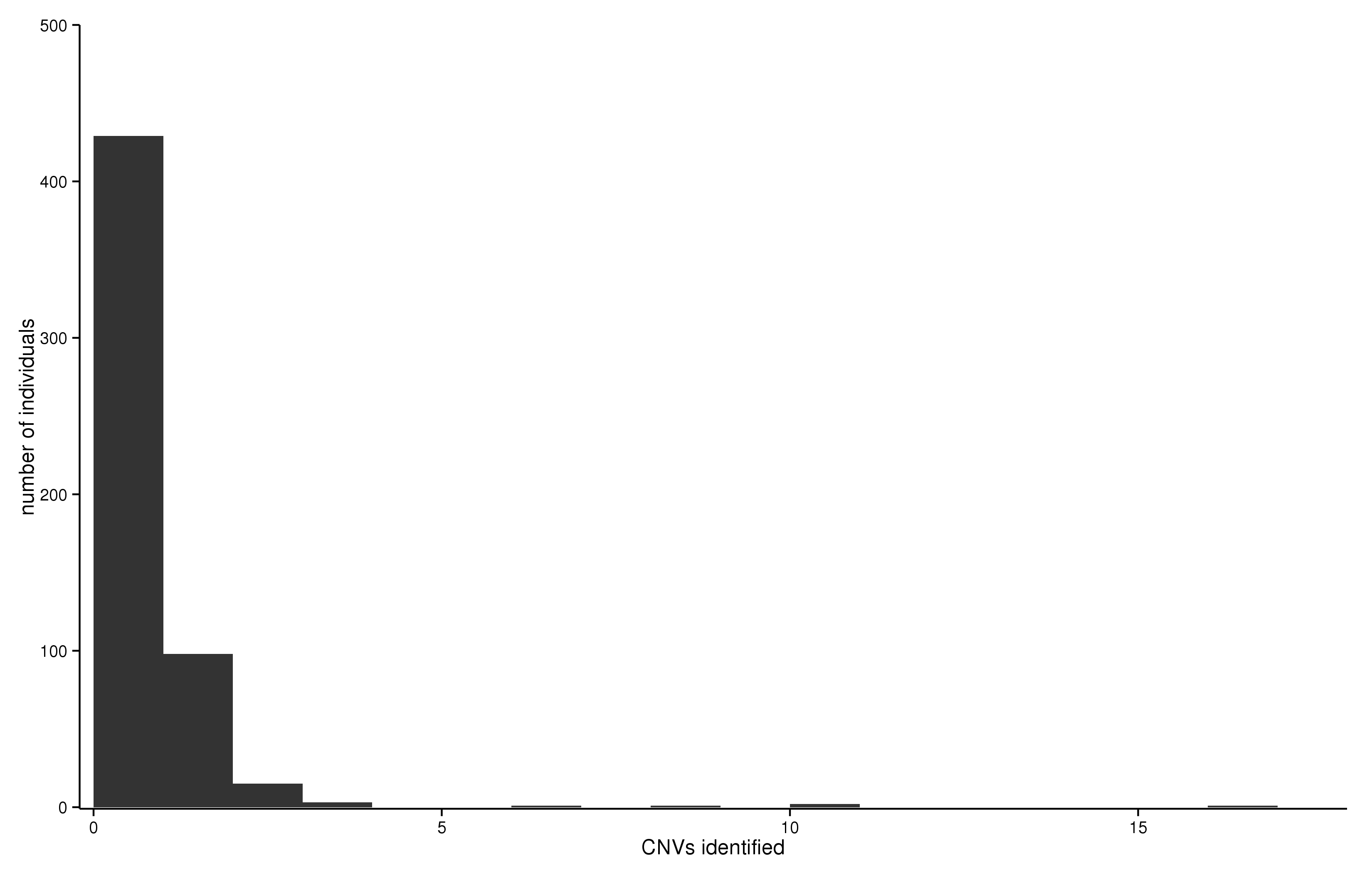


ExomeDepth

**
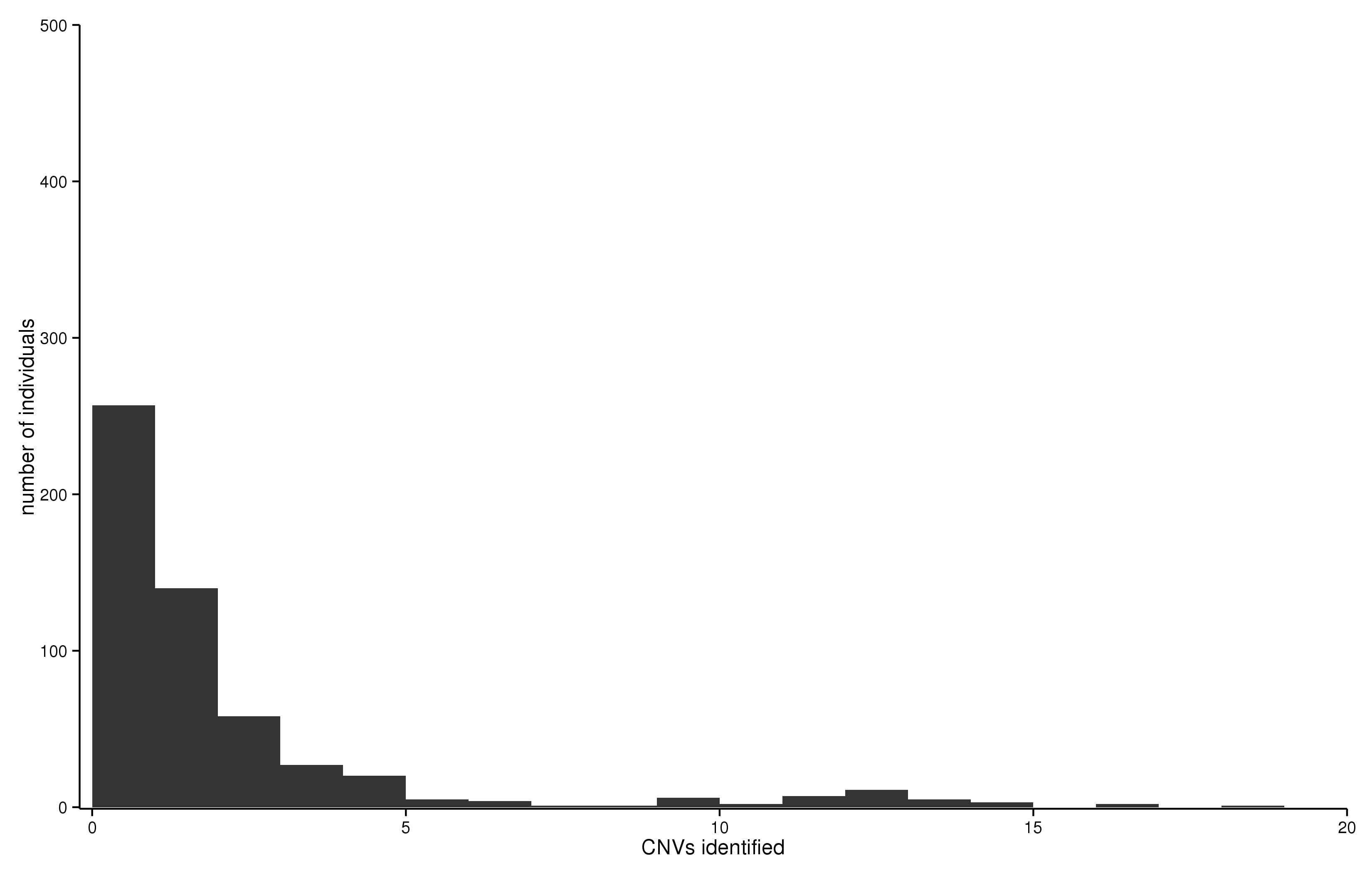
**

number of individuals

CoNVex

**
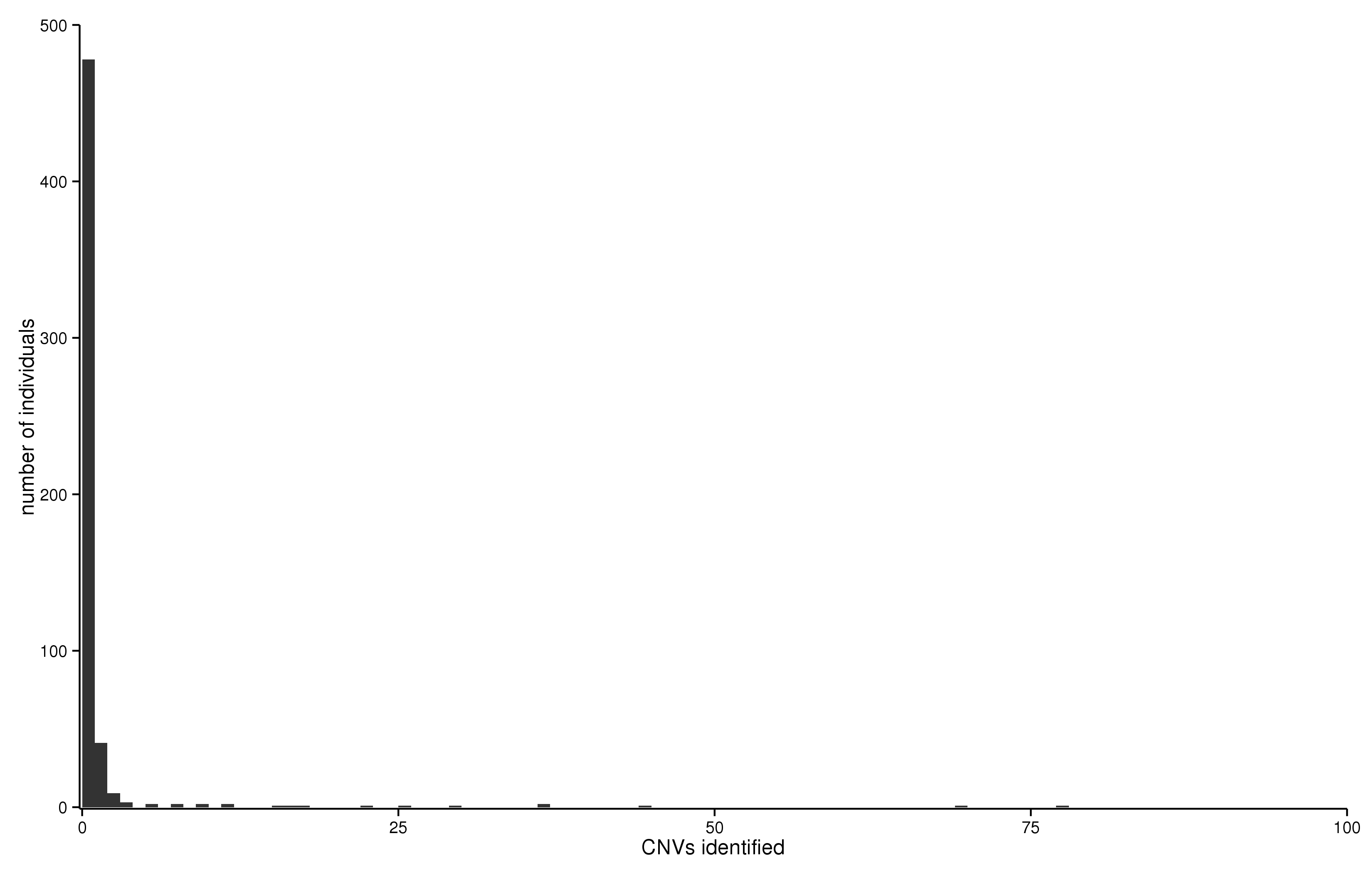
**

CoNVaDING

number of CNVs identified

**Figure S1. Histogram showing the number of copy number variants identified by each of the CNV detection algorithms for 550 individuals with inherited retinal disease.**

rpkm


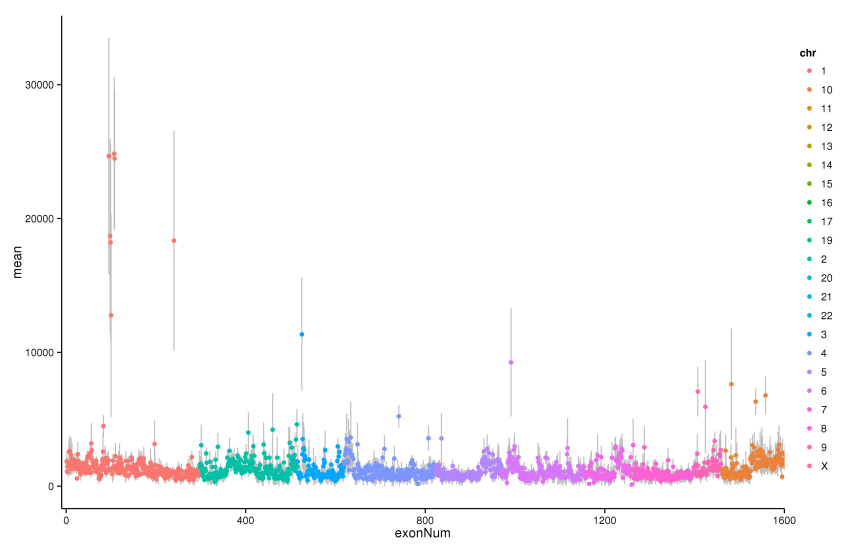

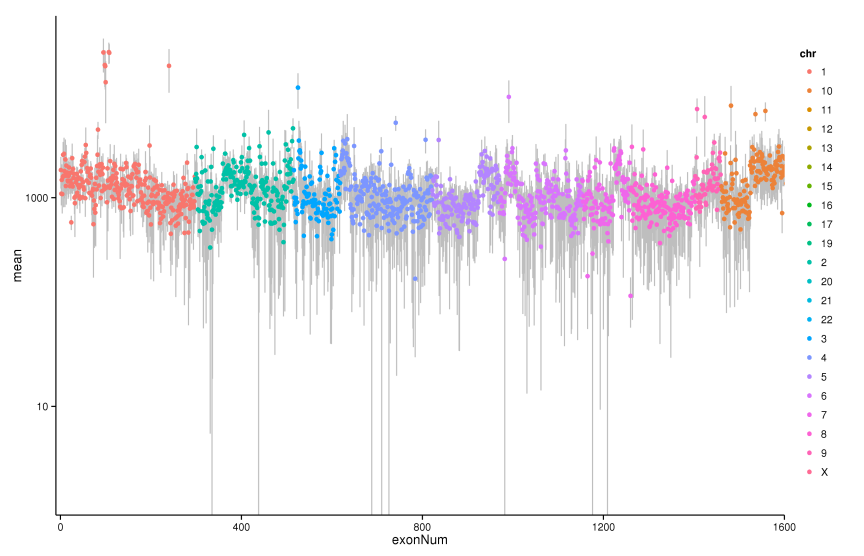

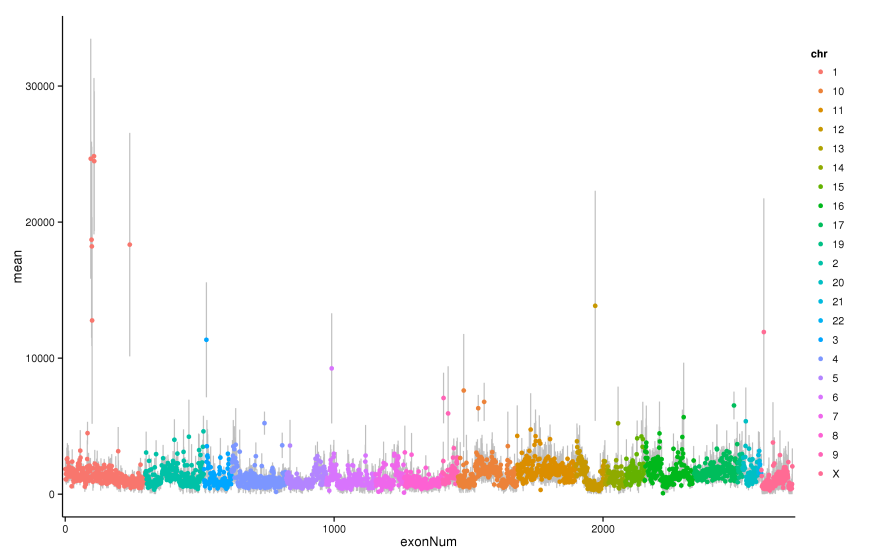

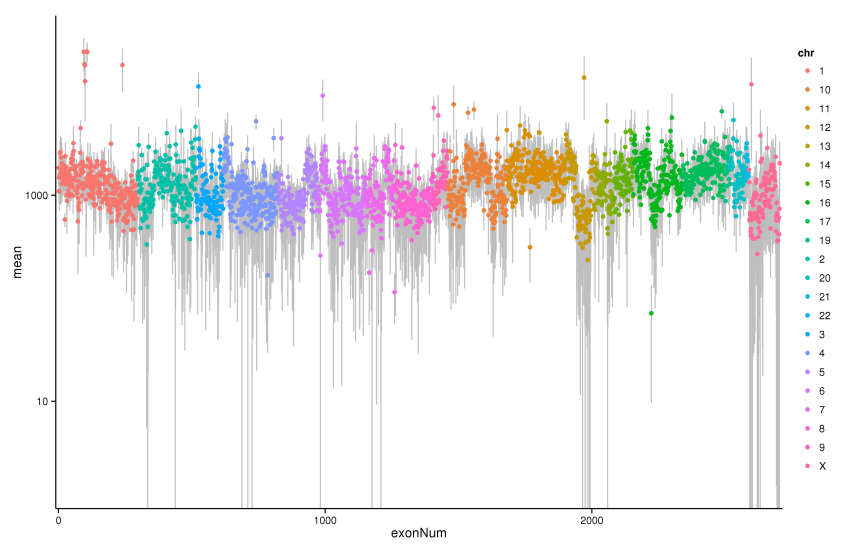


Exon Number (ordered by *hg19* coordinate)

**RDv2 (105 genes)**

**RDv3 (180 genes)**

**Figure S2. Average coverage profile for individuals analysed on each next-generation sequencing gene panel.** rpkm (*reads-per-kilobase-per-million*) values were calculated from properly paired reads with a quality value for alignment > 30. The upper and lower panels are plotted on a continuous and log10 scale, respectively. The change in dot colour indicates a transition to a new chromosome. 95% confidence intervals for the average rpkm are plotted as grey bars.


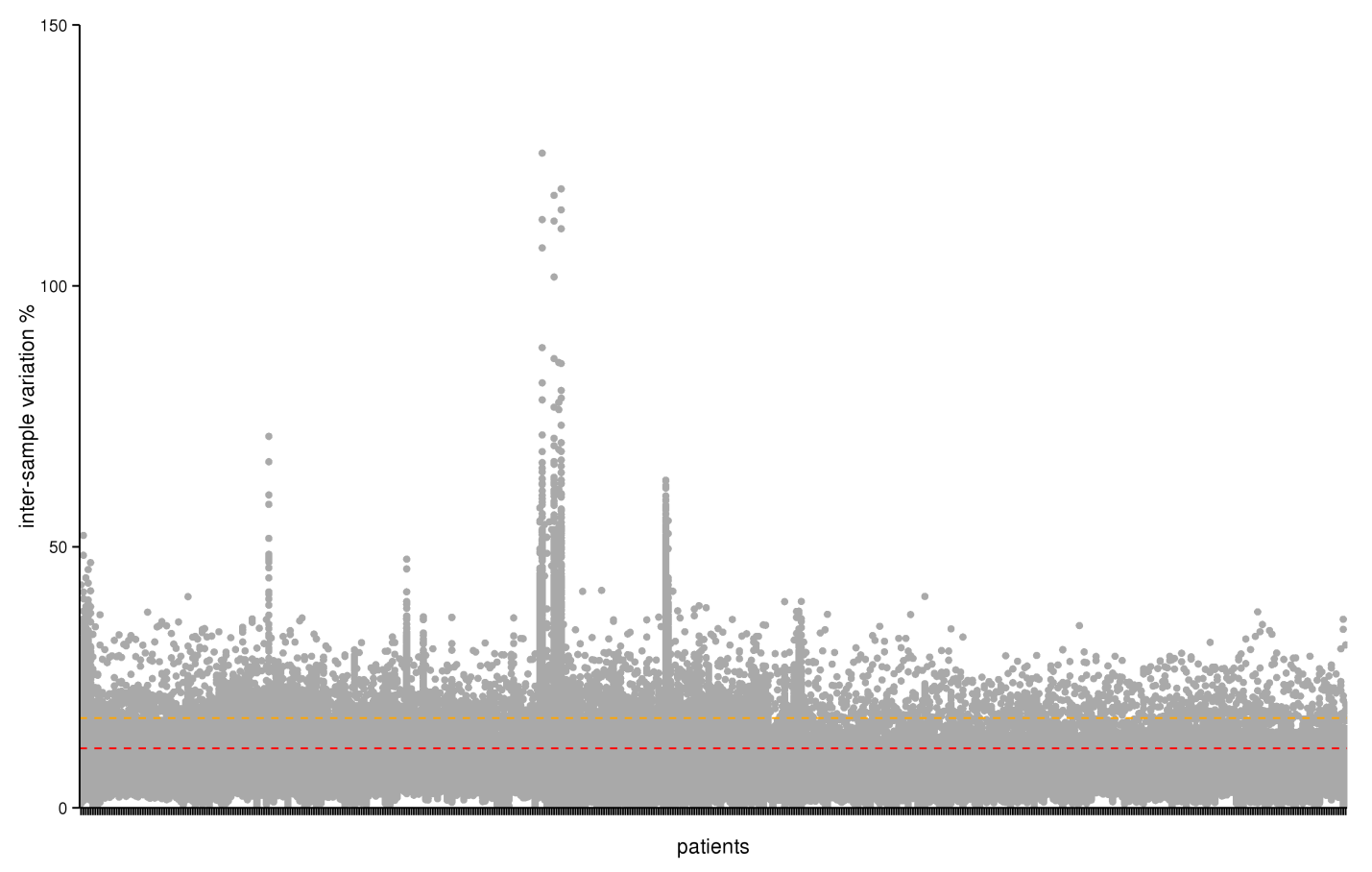
**Figure S3. Inter-sample variation observed for 550 patients for exons surveyed using gene panel NGS**. Solid circles represent the inter-sample variation calculated for each tested individual, defined as the coefficient of variation across reference samples selected by the ExomeDepth algorithm. We observe an average inter-sample variation of 5.83% (*n* Exons=1,224,686, median=5.25%, sd=3.28%). 95% of the exons surveyed demonstrate an inter-sample variation below 11.4% (*red horizontal line*) and this is consistent with an accuracy of 98.7% (*n* simulations=878). 99% of the exons surveyed demonstrate an inter-sample variation below 17.2% (*orange horizontal line*) and this is consistent with an accuracy of 98.2% (*n* simulations=954).

**
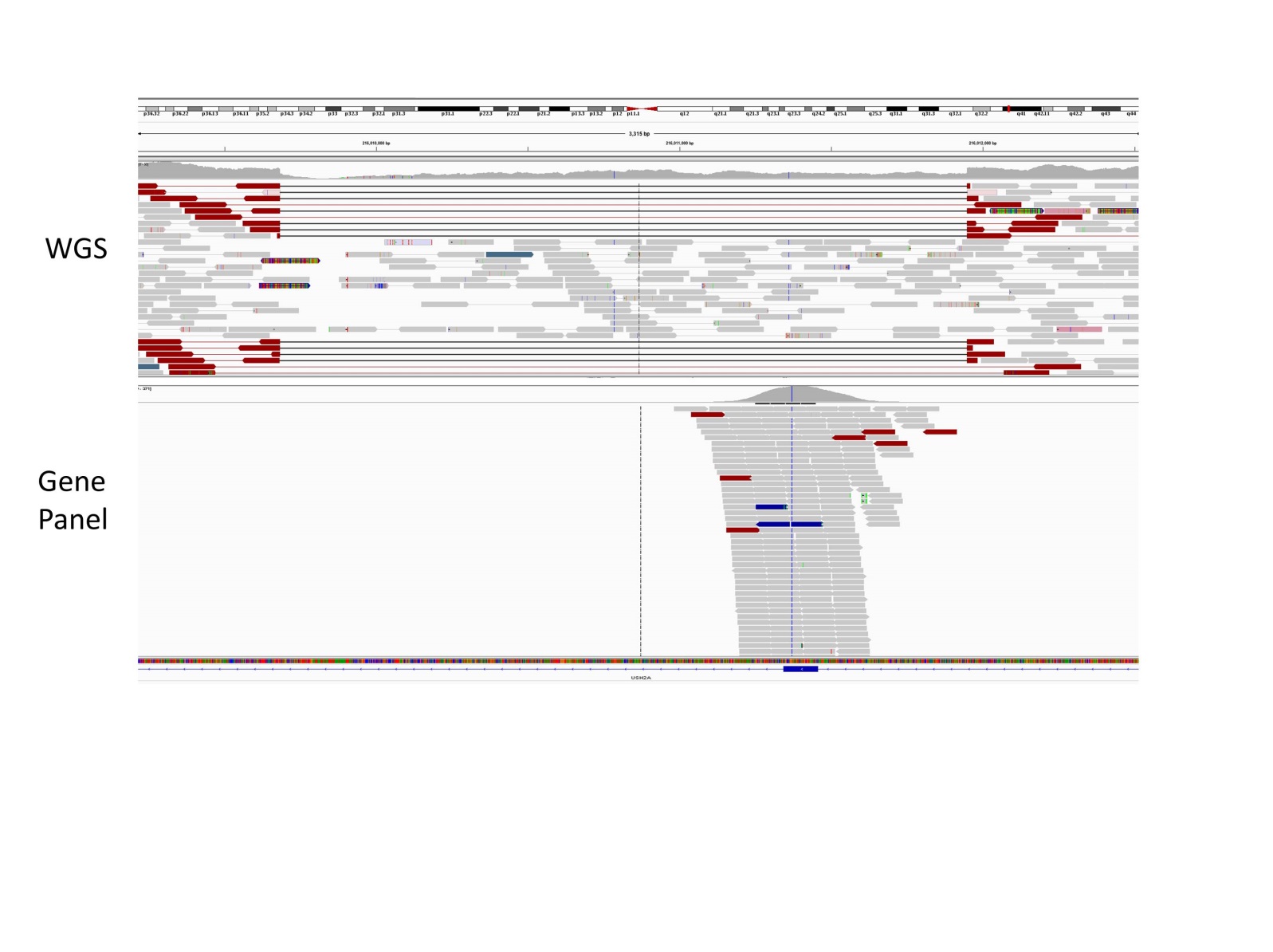
Figure S4. *USH2A* single exon deletion**. A deletion encompassing exon 47 of *USH2A* (NM_206933.2) was identified through ExomeDepth applied to gene panel next-generation sequencing datasets and through Manta applied to whole genome sequencing datasets generated through Illumina sequencing chemistry. The Integrative Genomics Viewer screenshots demonstrate the clear advantage of whole genome sequencing techniques to characterize large deletions encompassing exons with intronic breakpoints, with split-reads encompassing the identified deletion permitting detection at a single nucleotide resolution (chr1:216,009,683-216,011,948).


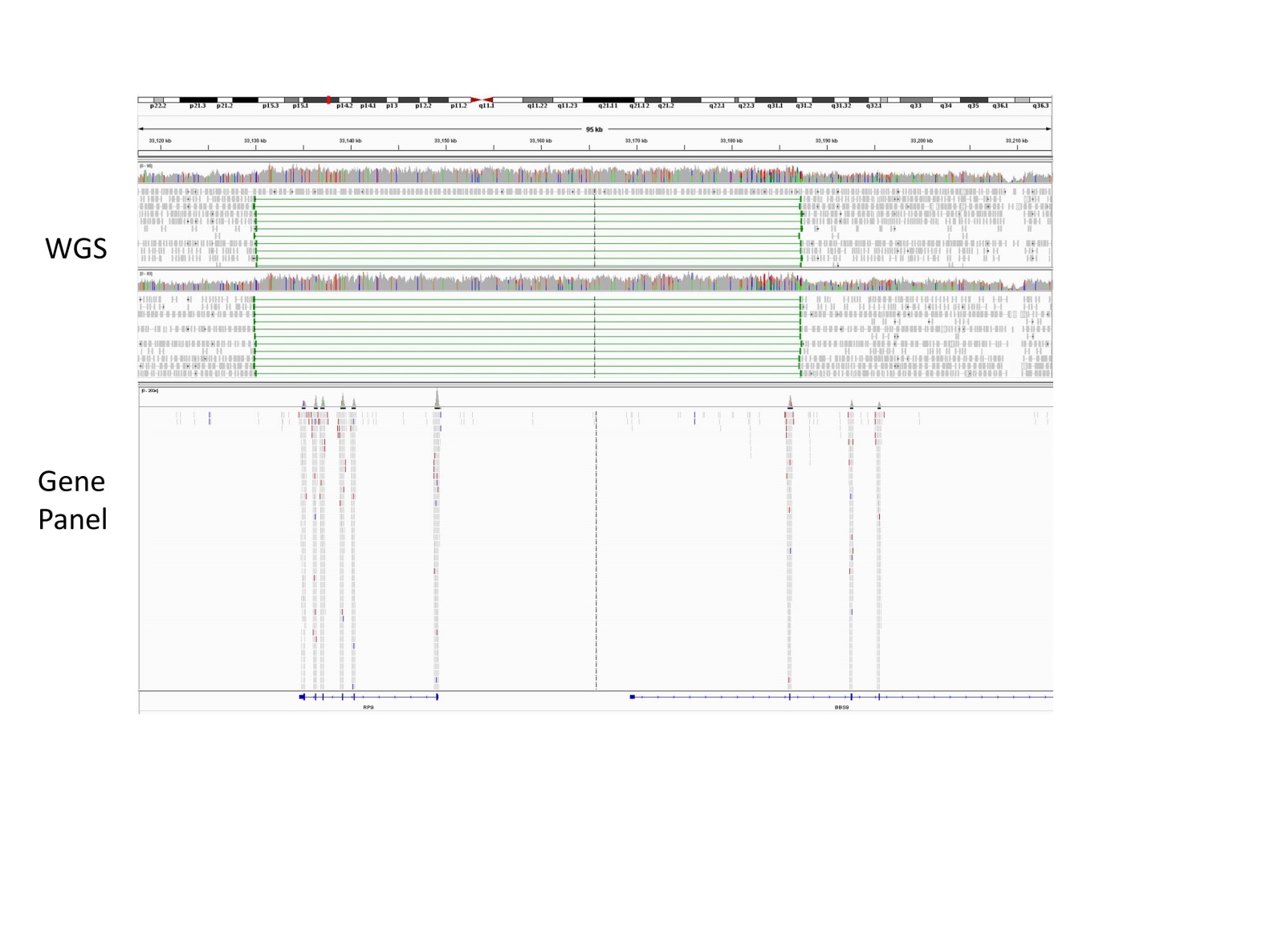


**Figure S5. Duplication event impacting *RP9* and *BBS9* identified in five unrelated individuals.** The duplication encompasses the complete coding region of *RP9* and the first coding exon of *BBS9*. The duplication was identified through ExomeDepth applied to gene panel next-generation sequencing datasets, and through Canvas applied to whole genome sequencing datasets generated through Illumina sequencing chemistry. The Integrative Genomics Viewer screenshots demonstrate the clear advantage of whole genome sequencing techniques to characterize large duplications encompassing exons with intronic insertion points. The top panel (*WGS*) shows reads generated for two individuals, with large insert sizes and everted read direction (*green*) in respect to the reference genome.

# Supplemental Case Study

A 15 year old boy was referred for genetic testing with a clinical indication of rod-cone dystrophy. The parents are consanguineous and there is no family history of inherited retinal disease. Surveillance of 176 genes identified two apparently homozygous variants. Paternal DNA was assessed for variant presence and zygosity. Maternal DNA was not available for familial segregation studies.

1. *PRPF31*: NM_015629.3: c.(?_-1)_(855+1_856-1)dup [4 copies confirmed]

Genomic rearrangements of *PRPF31* have been described previously as a cause of autosomal dominant retinitis pigmentosa.[^1^](#_ENREF_1) Duplications that partially impact the coding regions of *PRPF31* have been described as a cause of autosomal dominant retinitis pigmentosa.[^2^](#_ENREF_2) Mutations in *PRPF31* are known to display incomplete penetrance within families. The identified duplication is predicted to impact 7 coding exons of *PRPF31* (NM_015629.3). Droplet digital PCR confirmed that 4 copies of *PRPF31* exon 4 (NM_015629.3) are present in the affected individual’s DNA and that 3 copies of *PRPF31* exon 4 are present in the unaffected father’s DNA.

**Conclusion:** It is highly likely that the *PRPF31* duplication is present in a homozygous state in the boy clinically presenting with rod-cone dystrophy, although this has not been confirmed. This variant is currently considered to be of unknown significance. Future investigations to determine the location, phase and effect of this duplication on the expression of *PRPF31* will assist clinical interpretation.

1. *TULP1*: NM_003322.5 c.1047T>G p.(Asn349Lys) [homozygosity confirmed]

TULP1 c.1047T>G p.(Asn349Lys) has previously been reported in a homozygous state in patients with retinitis pigmentosa.[^3^](#_ENREF_3)^,^[^4^](#_ENREF_4) The Asn349 residue is located within the conserved C-terminal tubby domain and is highly conserved in several species.[^4^](#_ENREF_4) Segregation analysis through bidirectional Sanger sequencing determined that TULP1 c.1047T>G was in a heterozygous state in the DNA of the unaffected father.

**Conclusion:** The presence of TULP1 c.1047T>G in a homozygous state is consistent with a clinical diagnosis of autosomal recessive rod-cone dystrophy.

**1.** Sullivan LS, Bowne SJ, Seaman CR, et al. Genomic rearrangements of the PRPF31 gene account for 2.5% of autosomal dominant retinitis pigmentosa. *Investigative Ophthalmology & Visual Science.* Oct 2006;47(10):4579-4588.

**2.** Martin-Merida I, Sanchez-Alcudia R, Fernandez-San Jose P, et al. Analysis of the PRPF31 Gene in Spanish Autosomal Dominant Retinitis Pigmentosa Patients: A Novel Genomic Rearrangement. *Invest Ophthalmol Vis Sci.* Feb 01 2017;58(2):1045-1053.

**3.** Eisenberger T, Neuhaus C, Khan AO, et al. Increasing the Yield in Targeted Next-Generation Sequencing by Implicating CNV Analysis, Non-Coding Exons and the Overall Variant Load: The Example of Retinal Dystrophies. *PLoS One.* Nov 2013;8(11):18.

**4.** Kannabiran C, Singh H, Sahini N, Jalali S, Mohan G. Mutations in TULP1, NR2E3, and MFRP genes in Indian families with autosomal recessive retinitis pigmentosa. *Mol Vis.* 2012;18:1165-1174.
